# Effect of mastication on lipid bioaccessibility of almonds in a randomized human study and its implications for digestion kinetics, metabolizable energy, and postprandial lipemia^1^,^2^,^3^,^4^

**DOI:** 10.3945/ajcn.114.088328

**Published:** 2014-11-12

**Authors:** Myriam ML Grundy, Terri Grassby, Giuseppina Mandalari, Keith W Waldron, Peter J Butterworth, Sarah EE Berry, Peter R Ellis

**Affiliations:** 1 From the Biopolymers Group, Diabetes and Nutritional Sciences Division, King's College London, United Kingdom (MMLG, TG, PJB, SEEB, and PRE); the Institute of Food Research, Norwich Research Park, United Kingdom (GM and KWW); and the Department of Drug Science and Products for Health, University of Messina, Italy (GM).

**Keywords:** almonds, lipid bioaccessibility, mastication, mathematical model, microstructure

## Abstract

**Background:** The particle size and structure of masticated almonds have a significant impact on nutrient release (bioaccessibility) and digestion kinetics.

**Objectives:** The goals of this study were to quantify the effects of mastication on the bioaccessibility of intracellular lipid of almond tissue and examine microstructural characteristics of masticated almonds.

**Design:** In a randomized, subject-blind, crossover trial, 17 healthy subjects chewed natural almonds (NAs) or roasted almonds (RAs) in 4 separate mastication sessions. Particle size distributions (PSDs) of the expectorated boluses were measured by using mechanical sieving and laser diffraction (primary outcome). The microstructure of masticated almonds, including the structural integrity of the cell walls (i.e., dietary fiber), was examined with microscopy. Lipid bioaccessibility was predicted by using a theoretical model, based on almond particle size and cell dimensions, and then compared with empirically derived release data.

**Results:** Intersubject variations (*n* = 15; 2 subjects withdrew) in PSDs of both NA and RA samples were small (e.g., laser diffraction; CV: 12% and 9%, respectively). Significant differences in PSDs were found between these 2 almond forms (*P* < 0.05). A small proportion of lipid was released from ruptured cells on fractured surfaces of masticated particles, as predicted by using the mathematical model (8.5% and 11.3% for NAs and RAs, respectively). This low percentage of lipid bioaccessibility is attributable to the high proportion (35–40%) of large particles (>500 μm) in masticated almonds. Microstructural examination of the almonds indicated that most intracellular lipid remained undisturbed in intact cells after mastication. No adverse events were recorded.

**Conclusions:** Following mastication, most of the almond cells remained intact with lipid encapsulated by cell walls. Thus, most of the lipid in masticated almonds is not immediately bioaccessible and remains unavailable for early stages of digestion. The lipid encapsulation mechanism provides a convincing explanation for why almonds have a low metabolizable energy content and an attenuated impact on postprandial lipemia. This trial was registered at isrctn.org as ISRCTN58438021.

## INTRODUCTION

Evidence from epidemiologic and human metabolic studies has shown that the consumption of nuts such as almonds reduces a number of risk factors associated with noninfective disease, for example, type 2 diabetes, cardiovascular disease, and obesity (1–4). The behavior of almonds in the gastrointestinal tract may explain why almonds have these potential health benefits, notably a slow rate and limited extent of digestion of almond lipid and other macronutrients after mastication (5–7). These effects are strongly linked to the structure and properties of almonds, particularly the structural integrity of their cell walls (i.e., dietary fiber). Almond seeds are an energy-dense food, typically containing ∼50% of lipid, so they would be expected to elicit a relatively high postprandial lipemic response when ingested and to be associated with increased levels of obesity. However, previous work has revealed that a high proportion of lipid remains encapsulated in the cells of almond tissue and is therefore less available for digestion (6), leading to reduced energy absorption (8, 9) and a low postprandial lipemic response (5). These findings are reinforced by a recent study showing that the Atwater factors, used for estimating the metabolizable energy content of foods, overestimate the energy content of almonds by as much as ∼32% (8).

Variations in the size and structural characteristics of masticated plant foods are known to have a significant impact on nutrient release, digestion kinetics, gut hormone signaling, and other physiologic processes in the gastrointestinal tract (6, 9, 10). When mechanical stress is applied to edible plant tissue during mastication, the nutrient-rich cells may rupture or separate, depending on factors such as cell-cell adhesion and fracture properties of the cell walls (11). This behavior has important implications for nutrient release (i.e., bioaccessibility), which depends on the proportion of ruptured cells, relative to intact cells, in the plant tissue after mastication. Bioaccessibility refers to the amount of ingested nutrients released from a food matrix that becomes potentially available for digestion and/or absorption in the gastrointestinal tract. Almond cells rupture rather than separate when masticated, so their contents become potentially available for digestion (5, 6). The relation between particle size of masticated plant foods, which reflects the proportion of ruptured cells in the plant tissue, and nutrient release has received limited attention, with the exception of foods such as carrots (12–14).

We previously described a theoretical model for predicting lipid bioaccessibility in almonds, based on the dimensions of almond cells and geometrically defined particles (“cubes”) (15). In the current study, the model was applied to particle size distributions (PSDs)^5^ from masticated almonds, and the resulting bioaccessibility predictions were then compared with empirical data for lipid release. To obtain reliable predictions from the model, we needed to determine the PSDs of raw and roasted almonds masticated by human volunteers. In addition to this novel approach of modeling the first stage of human digestion, a detailed microstructural analysis of masticated almonds was performed to facilitate our understanding of how lipid is released from almond cells.

## MATERIALS AND METHODS

### Subjects

All mastication sessions took place in the metabolic unit facilities at King's College London, University of London. Of the 17 healthy adults recruited from the staff and students of King's College London, 15 completed the study [11 women and 4 men; mean age of 25.4 ± 5.8 y and BMI (in kg/m^2^) of 21.6 ± 3.7]. Previous studies investigating PSD under similar conditions have reported statistically significant differences in 10–13 subjects (9, 16–19). Therefore, on this basis, the number of volunteers recruited was 17 to allow for a 15–20% dropout. Exclusion criteria included allergy to almonds or related allergens (other tree nuts, celery, pears, apples, cherries, peaches, or parsley); incomplete dentition, other than unerupted wisdom teeth; any dental treatment in the past 3 months, except for a routine checkup; and current infectious disease. None of the volunteers included in the study showed any evidence of malocclusion and masticatory malfunction.

The study protocol was approved by the Research Ethics Committee of the North London's National Research Ethics Service (NRES 10/H0717/096), and written informed consent was provided by participants. The study visits started on May 2011 and were successfully completed in August 2011. This trial was registered at isrctn.org as ISRCTN58438021.

### Source and composition of test foods

Raw and roasted almond (*Amygdalus communis* L.; variety Nonpareil) kernels were produced by Hughson Nut Inc. and provided by the Almond Board of California. Given that whole almonds are mainly consumed in their natural (raw) or roasted form, both these types, designated NA (natural almond) and RA (roasted almond), respectively, were used in this study to estimate the impact of processing on the structure and behavior of the almond seed during mastication. The nutrient contents (percentage by weight of edible portion) of NAs and RAs were respectively as follows: moisture, 5.1% and 2.7%; ash (total minerals), 2.7% and 3.4%; protein (total nitrogen × 5.18), 20.1% and 20.7%; lipid (Soxhlet, hexane), 51.7% and 52.4%; available carbohydrates (mainly sugars), 4.6% and 4.8%; and dietary fiber, 11.0% and 10.6%. The nutrient contents, expressed as means of duplicates, are presented on a dry weight basis. The dietary fiber value, determined by using the method from the “AOAC International,” is a reflection of the cell wall content (mostly nonstarch polysaccharides) of the almond seeds. The lipid component of almonds is mainly found in parenchyma cells of the cotyledon tissue as small oil bodies with a diameter range of 1–5 μm (6, 20); see the Results section on microstructural analysis for details.

### Experimental protocol

The study was a crossover, single-blind study of 4 mastication sessions, which were randomly allocated by using computer-generated random numbers. The study investigators generated the random allocation sequence, enrolled participants, and assigned participants to interventions. Each subject attended a total of 4 sessions, 2 per form of almond, NA and RA, with at least 1 wk between each session. Each subject was blinded to the almond form and asked to masticate each almond sample (4–5 g) on 10 different occasions during each chewing session (i.e., 10 replicates, with each mastication occasion separated by a rest period of 2 min and rinsing of the mouth with water). For the first 2 replicates, the participants masticated and swallowed as normal, and the number of mastication cycles (counted cycles = *N*) as well as the mastication duration (duration of sequences = *T*) were recorded and averaged. The mastication frequencies were then calculated by dividing *N* by *T*. These values were used as guides for the subsequent expectorations (i.e., remaining 8 replicates). In previous studies investigating masticatory function and efficiency, the measure of mastication sequences, cycles, and frequency provided information on the individual mastication behavior (21). Such information is therefore useful in studies linking mastication to nutrient bioaccessibility; these parameters are expected to vary depending on the individual as well as the food and its physical properties (22–25).

During these tests, the participants masticated the sample until they reached *N* chews, at which stage they expectorated the contents of their mouth into individual preweighed plastic containers. They then rinsed their mouth with about 25 g water and emptied it into the previously used container to maximize recovery of the chewed almond samples. The samples were analyzed soon after collection except those used for lipid analysis, for which the almond boluses were stored at −20°C before being processed. The primary outcome measure was the PSDs of the boluses, and the secondary outcome measure was microstructural analysis of the boluses.

### Particle sizing

A wide range of techniques has been used for determining the average particle size and PSDs of masticated foods. In previous reports, mechanical sieving, laser diffraction, image analysis, and optical scanning methods have been used on natural (13, 17, 18, 26, 27) as well as artificial (28–30) foods to evaluate inter alia masticatory efficiency. For almonds, the method predominantly employed by other research groups has been mechanical sieving (9, 16, 19, 31, 32). However, this method is limited by the amount of information that can be obtained for PSDs (24). In the present study, mechanical sieving and laser diffraction were compared and subsequently combined to cover the whole PSD. These methods were selected to cover the broad range of the PSDs of almond boluses and also to facilitate comparison with other research groups that have employed similar techniques (9, 16, 18).

#### Mechanical sieving

For each of the 15 subjects, 2 replicates of masticated samples were combined (∼10 g of almond boluses) and loaded on a stack of sieves with 10 aperture sizes: 3.35, 2.0, 1.7, 1.0, 0.85, 0.50, 0.25, 0.125, 0.063, and 0.032 mm (Endecott test sieve shaker). A nylon mesh with a 0.020-mm aperture was also placed between the sieve base and the 0.032-mm sieve to allow comparison with the laser diffraction. The expectorated samples were then washed with deionized water, shaken for 15 min, and washed again, thus ensuring that the particles were properly sieved. They were then dried in the forced-air oven at 56°C for 6 h as previously described (9, 16). The base was left to dry at 100°C overnight, which permitted the total evaporation of the water. The sieves were weighed before loading the sample and then again after having been dried in the oven. The dried fractions retained on each sieve and the base were expressed as a percentage of the weight of almonds before mastication.

#### Laser diffraction

The sample preparation was similar to the process already described for mechanical sieving. Thus, 2 of the masticated samples (replicates) were combined and poured onto a sieve with a 1700-μm aperture. The sieve was placed on top of a sieve base covered with a nylon mesh (aperture of 20 μm) and washed with deionized water. Once the water had passed through the mesh, the retained particles were transferred into a 250-mL glass bottle by washing them off the mesh with deionized water. Removing particles of sizes >1700 μm and <20 μm prevented, for the former, obstruction of the instrument (upper size limit between 1500 and 2000 μm, depending on particle shape) and, for the latter, interference with the measurements, because particles of these sizes correspond only to cell wall fragments and intracellular contents (e.g., oil droplets). These materials were examined by light microscopy, and there was no evidence of intact cells (data not shown).

The protocol used for the particle size measurements with the laser diffraction was adapted from previous work (19). This method involved loading the samples into a Malvern laser diffraction particle sizer 2000 via a dispersant unit (Hydro 2000G) filled with water (Malvern Instruments Ltd.). Before loading, each sample was divided into several approximately equal quantities, and consecutive 10-second measurements were taken for each of these subsamples. The set of measurements obtained was averaged to give the PSD for the whole sample. The speeds of the stirrer and the pump were 700 and 1175 rpm, respectively. These settings were selected because under these conditions, the samples were well dispersed into water and therefore showed no aggregation and consistently low intra- and intersample variation of samples produced from each individual subject (i.e., average CV of 6%, and the laser obscuration did not fluctuate over time). The diffraction data were analyzed by using the Mie diffraction method, which is used for accurately measuring the light-scattering behavior of spherical particles over a large size range (0.02–2000 μm) (Malvern Instruments Ltd.). The proportion of sample in each particle size interval was reported as volume percentage of the whole PSD.

### Determination of lipid bioaccessibility

#### Predictions from the theoretical model

The original theoretical model (15) was applied to particle size distributions from masticated raw and roasted almond boluses to provide predictions of bioaccessibility from samples containing heterogeneous particle sizes. For the current study, this model was adapted to allow predictions of bioaccessibility by using heterogeneous particle sizes of masticated raw and roasted almond boluses. Data obtained from the 2 particle sizing methods, sieving and laser diffraction, were used for model predictions. The original model (Equation *1*) predicts the fraction of lipid released from particles of almond cotyledon tissue with a specific particle edge length (particle size, *p*) and average cell diameter (*d*), with *d* being ∼35 μm for almond parenchyma cells containing lipid (15):


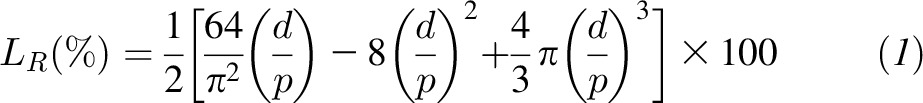


where *L*_R_ is the percentage of lipid release.

The initial model was constructed on the basis that the almond particles were theoretical cubes for 2 reasons, first that it simplified the development of the model and second that cubes were used as an experimental tool in our previous in vitro and in vivo digestibility studies (7, 15). To predict lipid release values from the mastication size data by using Equation *1*, we needed to transform these data into particle edge lengths. However, the laser diffraction method generated particle size values for masticated almonds expressed as a volume-equivalent sphere diameter, which is the diameter of a sphere with the same volume as the particle. The sphere diameters (*D*) were therefore converted into particle edge lengths (*p*) with the following equation:


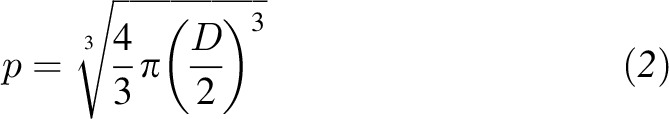


It was also assumed that only the cells through which the fracture plane passes were ruptured (i.e., the surface of ruptured cells created by fracturing the almond) and therefore released their contents, as observed previously (6). The sieve particle sizes were also converted into particle edge lengths with Equation *2*. The mathematical model was used to calculate lipid bioaccessibility for each particle size (*p*) and then multiplied by the weight percentage of that particle size fraction in the complete bolus to give lipid bioaccessibility for the bolus.

The weight percentages of 4 fractions, with particle size ranges of 0.02–1.7 mm, 1.7–2.0 mm, 2.0–3.35 mm, and >3.35 mm, were calculated relative to the total weight retained by the sieves. The percentage weight values of the different subfractions within the 0.02–1.7 mm size range were estimated by using the laser diffraction data. The values for each fraction were then combined to give the predicted lipid release, expressed as a percentage, for each bolus (*L*_T_) produced by the volunteers (see **Supplemental Table 1** for example calculations).

#### Bioaccessibility analysis by solvent extraction method

The lipid contents of the original NAs and RAs and the corresponding masticated almonds were determined to obtain the amount of lipid that had been released during the chewing process. Four volunteers masticated a typical portion size of almonds (28 g, ∼4.5 g per mouthful) and expectorated it in a similar manner to that described above. This amount was chosen so as to provide enough material for lipid determinations to be performed reliably and with good precision. Following centrifugation of the expectorated samples, the liquid phase of the collected sample was removed; the remaining particles were then dried, weighed, and analyzed. Lipid extraction was performed with hexane as solvent according to the Soxhlet extraction method (7). Lipid bioaccessibility was estimated by calculating the difference between the total lipid content of the original almond samples and the lipid content of the almonds after mastication but appropriately adjusted to account for the loss of almonds in the mouth. The results of lipid content were expressed as a percentage of dry weight. The experimental data were then compared with the lipid bioaccessibility values obtained from the theoretical model by using particle size data of masticated almonds generated from the same 4 volunteers.

### Microstructural analysis

Masticated samples were first left in a 2.5% (vol:vol) glutaraldehyde solution for 2 wk before being postfixed in 2% (wt:vol) osmium tetroxide. The samples were dehydrated in a graded ethanol series (10%, 20%, 30%, 40%, 50%, 60%, 70%, 80%, 90%, and 100%, by volume) and then placed in a series of solutions containing propylene oxide and 100% ethanol in the following proportions: 1:1, 2:1, and 1:0. A Spurr low-viscosity resin (London Resin Company Ltd.) was used to embed the masticated almond particles. Thin sections (70 nm for transmission electron microscopy) and semi-thin sections (1 μm for light microscopy) of the embedded samples were cut with a Diatome diamond knife (Leica Microsystems Ltd.). The sections were transferred onto a drop of water on a glass slide and dried on a hot plate. They were then stained with 1% (wt:vol) toluidine blue in 1% (wt:vol) sodium borate. The slides were viewed under either the optical Zeiss Axioskop 2 mot plus microscope (Carl Zeiss Ltd.) or the Tecnai T12 transmission electron microscope (FEI Europe) fitted with an AMT camera system. Samples intended for scanning electron microscopy were treated as described previously (6) by using critical point drying in a Polaron E3000 CP Drier (Quorum Technologies). The masticated almond tissues were mounted on stubs, coated with gold in a Polaron E5100 sputter coating unit, and viewed in a JEOL 25SM and a Philips 501 scanning electron microscope (FEI Company).

Nile red (1 mg/mL in dimethyl sulfoxide) was also used to identify lipids in fresh particles of almond samples, which were then examined immediately on the light microscope (Zeiss Axioskop 2 mot plus microscope).

### Statistical analysis

The data were analyzed by using SPSS version 20.0 (SPSS Inc.). For all tests, the significance level was set at *P* < 0.05 (2-tailed). All data were normally distributed (analyzed by using the Shapiro-Wilk test and Normal Q-Q plots); they are expressed as means ± SEMs. Repeated-measures ANOVA was used to assess the differences in PSDs between replicates (i.e., visits 1 and 2) and almond form and also differences in lipid release between the 2 methods (i.e., Soxhlet and mathematical model). Differences in masticatory parameters, particle size, and lipid release between the almond forms were tested by Student's paired *t* test.

## RESULTS

### Masticatory parameters

A total of 17 subjects were randomly assigned to the mastication sessions and commenced the study, 2 subjects withdrew because of time constraints, and the remaining 15 subjects completed all 4 mastication sessions. The results (means ± SEMs) showed no statistically significant differences in the number of mastication cycles and mastication frequency between the 2 forms of almond, NAs and RAs. Thus, values for the number of mastication cycles for NAs and RAs were 34.4 ± 3.9 and 33.1 ± 3.6, respectively, and for mastication frequency, the values were identical (i.e., 1.4 ± 0.05 s^−1^) for both NAs and RAs. Only the duration of the mastication sequences was statistically different (*P* < 0.05) between the almond forms, although this difference was relatively small, as seen by the mean values of 25.4 ± 2.72 s and 23.3 ± 2.40 s for NAs and RAs, respectively.

### Particle sizing of the masticated samples

**FIGURE 1:**
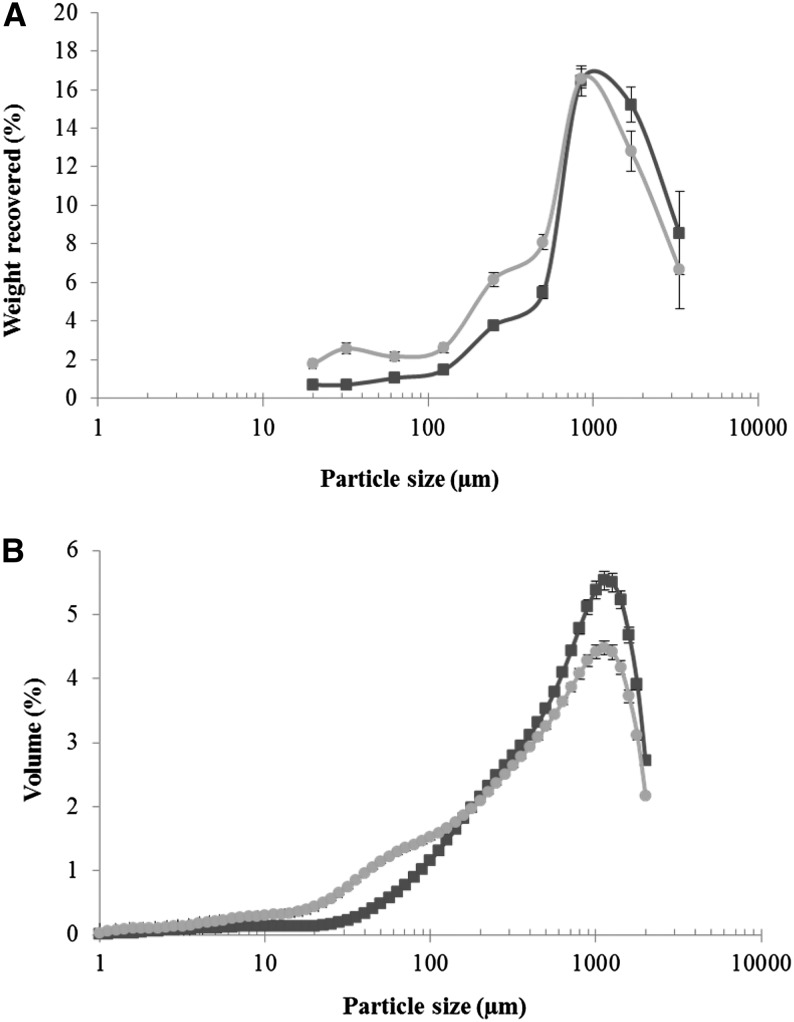
Particle size distributions of masticated almonds were measured by mechanical sieving (A) and laser diffraction (B); NA (dark-gray line) and RA (light-gray line) boluses. Size data are presented on a log scale plotted against percentage weight recovered (A) or percentage volume (B). Some sieve fractions with similar sieve apertures were combined (850 with 1000 μm and 1700 with 2000 μm), so that the total number of experimental points shown in the figure is 9, which also includes the 20-μm nylon mesh “sieve.” Student's paired *t* test indicated significant differences (*P* < 0.001) in particle size at all size fractions found between raw and roasted almonds, except for sizes 850 and 1000 μm and sizes 141, 159, 178, and 200 μm for sieving and laser methods, respectively. Individual experimental points on the size distribution profiles are means ± SEMs (*n* = 15). NA, natural almond; RA, roasted almond.

Each form of almond, collected on different days, was measured for each participant by both sizing methods, each of which has some methodologic limitations. We observed that, compared with mechanical sieving, laser diffraction was a more efficient, reproducible (as shown by the small error bars in **Figure 1**), and less time-consuming method. One advantage of mechanical sieving is that it provided a size distribution over a wider range of sizes compared with the laser method, albeit with poorer size resolution. Problems of sieve damage (especially at low aperture size) and particle aggregation were also experienced with mechanical sieving, whereas laser diffraction was not affected by such deficiencies.

#### Mechanical sieving

When the contents of the sieve base (i.e., containing only fragments of cells and cell walls and isolated intracellular components such as oil droplets) were included in the calculation, the NA and RA boluses had a total percentage recovery of 85.4 ± 1.5% and 89.5 ± 1.5%, respectively. The weight of masticated almond retained on the sieves, presented as a percentage of the original weight of the almond, was plotted against the aperture size of each sieve. The average PSDs for NAs and RAs are shown in Figure 1A. Sieve PSDs are usually measured by using a systematic mathematical progression in sieve aperture size; therefore, the fractions from the 1.7- and 2.0-mm sieves were combined, as were those from the 0.85- and 1.0-mm sieves, so that aperture size roughly doubled at each step. Repeated-measures ANOVA, with size as a factor, revealed significant differences in PSDs between the raw and roasted almonds (*P* < 0.05). Student's paired *t* test showed significant differences (*P* < 0.001) in particle size at all the size fractions between the raw and roasted almonds, except at size fractions 850 and 1000 μm, in which the 2 PSD curves overlapped. Therefore, the proportion of large particles (1700 to >3350 μm) was greater for NAs than for RAs, whereas the opposite was observed at the lower particle range (20 to <1700 μm), so the masticated roasted samples contained a higher proportion of small particles. This result is in agreement with data from a chewing study that used a similar sieving method (29); in our study, ∼60% and 24% of the particles from raw almonds, obtained with mechanical sieving, had particle sizes <500 μm and >1700 μm, respectively. Similar results were obtained for roasted almonds, with 64% of particles <500 μm and 20% >1700 μm.

#### Laser diffraction

The average PSDs of NAs and RAs obtained by laser diffraction are shown in Figure 1B. All PSDs were multimodal and broad and similar to the distributions obtained by mechanical sieving, except that the laser method does not include sizes at the high end of the distribution because of an upper size limit between 1500 and 2000 μm. Intersubject variation was relatively small (i.e., pooled CVs were 12% for NAs and 9% RAs). Student's paired *t* test indicated significant differences (*P* < 0.001) in particle size at all size fractions of the distributions between the 2 almond forms, apart from the size fractions 141, 159, 178, and 200 μm, in which the 2 PSD curves overlapped.

The data indicate that 47% and 56% of the NA and RA particles, respectively, have a size <500 μm. However, the laser measurements did not include particles >1700 μm because of size limits, as explained in Materials and Methods. In view of the reliability of the particle size data obtained from the mastication study, by using the 2 different sizing methods, we were justified in incorporating these data into the theoretical model for predicting lipid bioaccessibility.

### Lipid bioaccessibility determined by the theoretical model and solvent extraction

Lipid bioaccessibility was predicted from the theoretical model by using all the particle size data obtained from the mastication study (*n* = 15). The predicted mean lipid bioaccessibility values for NAs and RAs were 8.4 ± 0.32% and 11.1 ± 0.29%, respectively; statistically significant differences were found between these 2 almond forms (*P* < 0.001). The predicted lipid bioaccessibility ranges were 6.4–9.9% for NAs and 8.6–12.5% for RAs, reflecting the slightly increased proportion of small particles in the PSD of the RA form.

Lipid bioaccessibility of the almonds masticated by the human volunteers (*n* = 4) was also determined by using solvent (hexane) extraction. The results obtained from this experimental method are in close agreement with the predicted data from the theoretical model, with bioaccessibility values of approximately 8% and 11% for NAs and RAs, respectively (**Table 1**). The model indicated a threshold particle size value (*p*) of approximately 56 μm for almonds, which is the point at which no more intact cells are present in the particle, based on an average cell size of 35 μm. Therefore, to obtain 100% release, all the particles would have to be 56 μm or smaller. This is not the case with masticated almonds, and even almond flour (average particle size, 250 μm) has a predicted lipid release of ∼40%.

**TABLE 1:** Percentage of lipid release of masticated NAs and RAs estimated by the mathematical model using particle size data or measured by the Soxhlet solvent extraction method^1^

	Soxhlet, %		Mathematical model, %	
Volunteer	NA	RA	NA	RA
1	5.9	11.1	9.4	11.2
2	8.6	12.9	7.5	11.7
3	7.8	12.5	7.2	10.9
4	9.1	8.1	9.9	11.4
Mean ± SEM^2^	7.9 ± 0.70	11.1 ± 1.09	8.5 ± 0.67	11.3 ± 0.17

1 *n* = 4 volunteers. NA, natural almond; RA, roasted almond.

2 Significant differences between NA and RA (*P* < 0.05) as calculated by Student's paired *t* test were found, but no differences were found between the experimental and theoretical methods (repeated-measures ANOVA).

### Microstructure of masticated almonds

**FIGURE 2:**
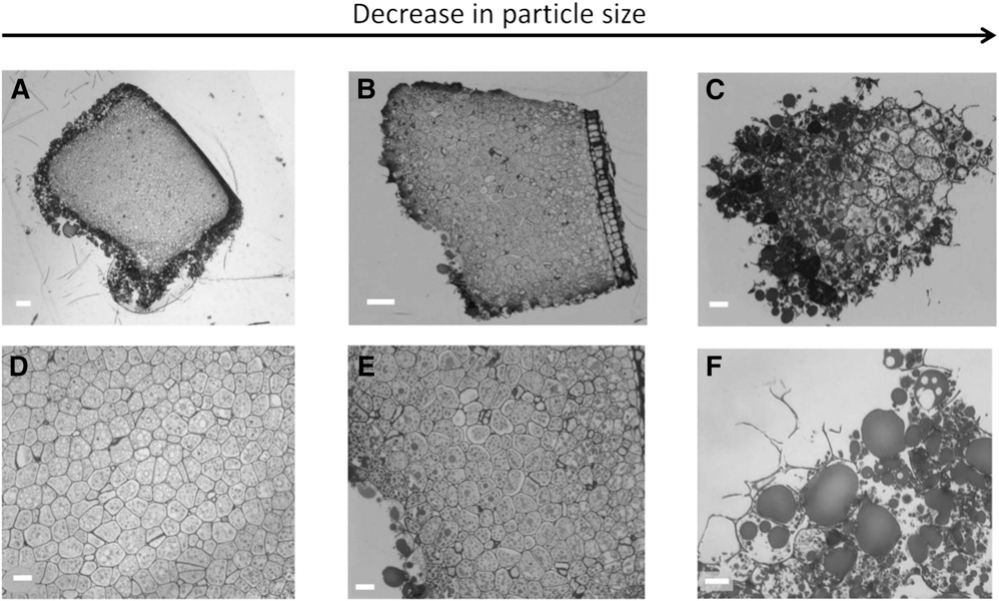
Light microscopy images of masticated NAs: whole particles of decreasing size (A, B, and C), parenchyma cells located in the center of the particles (D and E), and cells situated at the edge of the particles (F). Note the presence of coalesced lipid droplets (C, E, and F). Scale bars: A, 100 μm; B, 50 μm; C–E, 20 μm; F, 10 μm. Approximate sizes of NA particles: A, 1200 μm; B, 500 μm; C, 250 μm. NA, natural almond.

**FIGURE 3:**
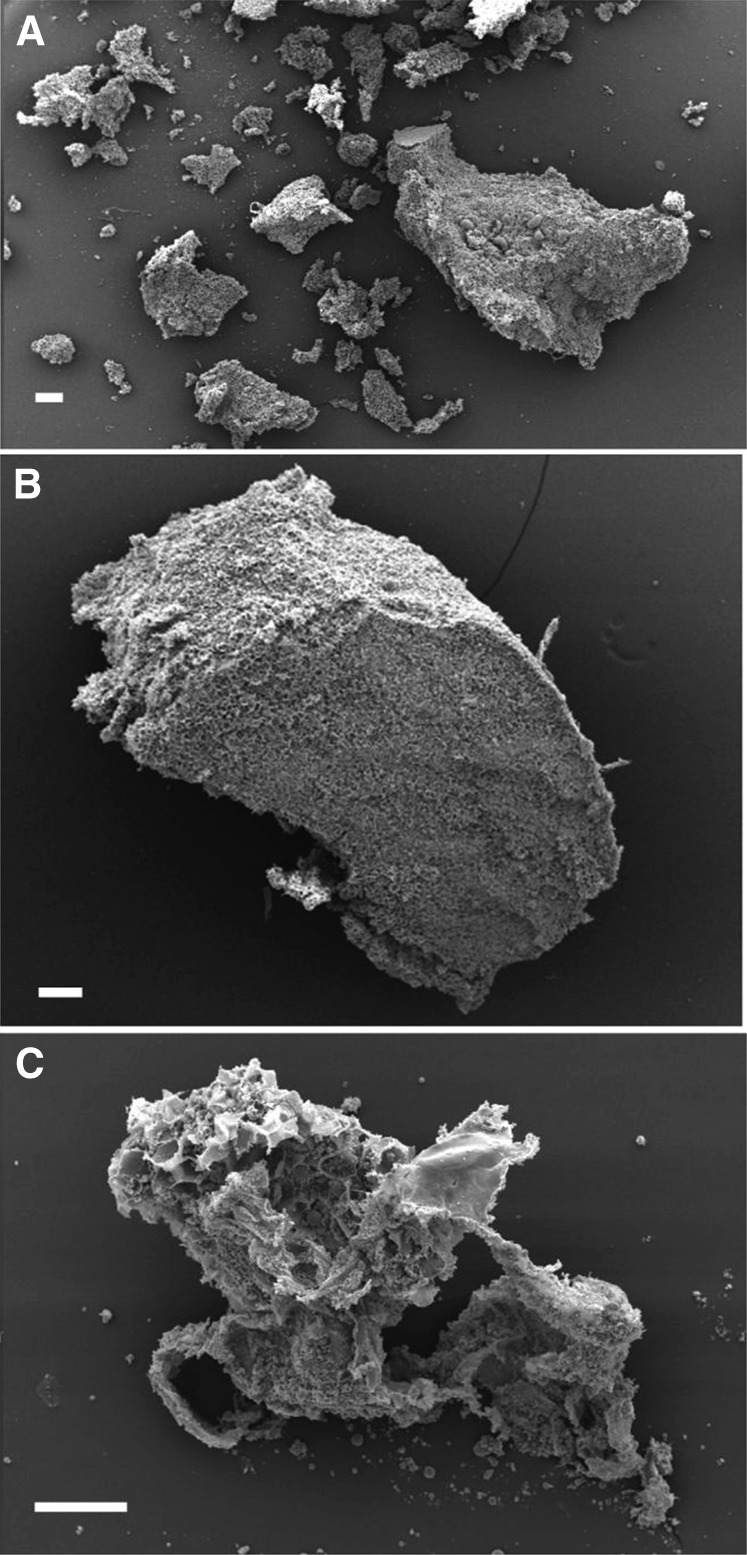
Scanning electron microscopy images of particles from masticated NAs. Scale bars: A and B, 200 μm; C, 100 μm. Approximate sizes of NA particles: B, 2000 μm; C, 550 μm. NA, natural almond.

**FIGURE 4:**
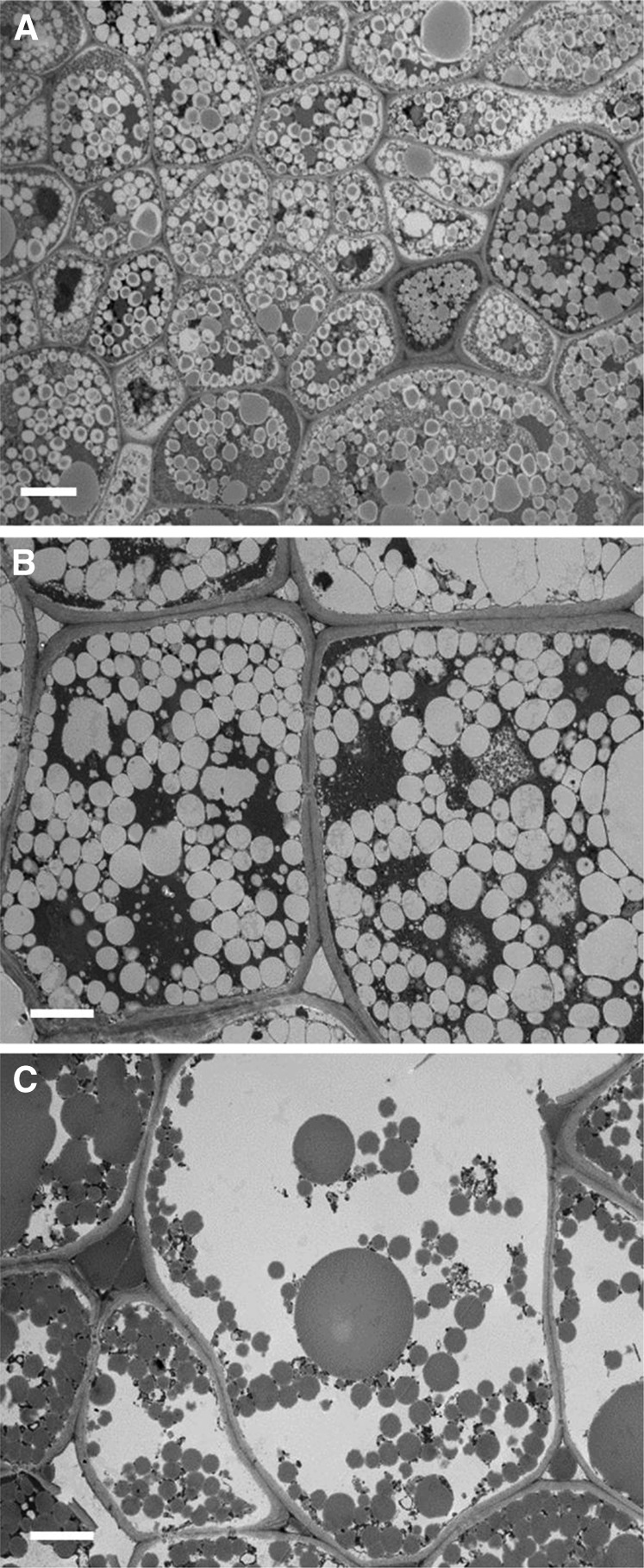
TEM images (A and B) of masticated NAs show intact cells and their content. The TEM image in panel C shows ruptured cells at the surface of the masticated NA particle; note the coalesced lipid bodies. Scale bars: A, 6 μm; B and C, 5 μm. NA, natural almond; TEM, transmission electron microscopy.

**FIGURE 5:**
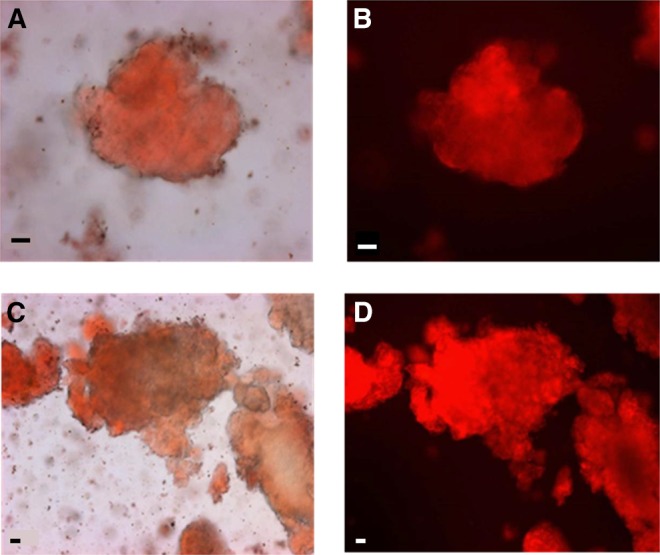
Light microscopy images of masticated natural almonds stained with Nile red indicating the presence of lipid. Scale bars: A–D, 20 μm.

The microstructural characteristics of masticated almonds (**Figure 2**) show that the lipid-rich parenchyma cells appear to remain largely intact, not just in the center of the particle but also located in cells immediately beneath the fractured surface. Thus, extensive cell breakage was observed mainly at the fractured surfaces of relatively large particles (e.g., sizes ∼1200 μm and 500 μm in Figure 2A and B). Moreover, there was little or no evidence of cell separation in these masticated particles. However, in particles of smaller size (∼250 μm), there was evidence of significant levels of cell distortion and rupture in all areas of the almond particle, not just at the fractured surface (Figure 2C). Scanning electron microscopy images provide further evidence of the apparent greater damage caused by chewing in the smaller almond particles (**Figure 3**). One possible explanation of this is that small particles may have received a larger number of deformations (chews) during mastication, potentially leading to greater structural damage to the cellular tissue. The lipid-rich parenchyma cells were tightly packed together, but much less so for some of the small particles, thus creating a compact tissue matrix that makes the diffusion of molecules (e.g., lipase) and water extremely difficult, as illustrated by the centers of the particles remaining unstained (Figure 2). The micrographs (Figure 2 and **Figure 4**) clearly show that most of the nutrients remained encapsulated in their original form inside the cells. These intracellular inclusions are mainly lipid bodies, as demonstrated by Nile red staining (**Figure 5**). The relatively uniform (“spherical”) microstructure of the oil bodies can be distinctly seen in transmission electron microscopy images in Figure 4A and B.

The cells located at the surface of the particles were ruptured and intracellular contents exposed to the external environment, although some of the nutrients, including lipids, were still present and thus not removed by saliva at the fractured surface (Figures 2C, E, and F and  4C). However, when masticated, tissue rupture appeared to occur unevenly within the almond particle, and fissures running from the fractured surface of almond particles into the underlying core tissue were observed (Figure 2C and F); some of these fissures created new particles that were eroded from the particle surface (Figure 2F). These fissures seemed to be more frequent in the small particles relative to large ones.

## DISCUSSION

To study the mechanisms of nutrient digestion and how this multiphase process is linked to absorption and postprandial metabolism, we need to improve our understanding of the disassembly of complex foods and nutrient release in the mouth and other sites of the digestive system. This approach has been used in the current study to obtain accurate and reproducible data on lipid bioaccessibility of almonds following mastication and to understand the mechanisms of lipid release from almond tissue during oral processing.

Diets high in fat are usually considered detrimental to health; therefore, individuals may avoid lipid-rich plant foods, such as almonds, despite the recognized health benefits associated with their consumption (2, 33). These benefits may be partly linked to the restricted digestion and absorption of fat and energy in almonds. Indeed, it has been reported recently that the energy content of almonds has been significantly overestimated when using Atwater factors (8). One crucial aspect previously overlooked in many nutrition studies is the complex behavior of food materials in the gastrointestinal tract. It is now well recognized that changes in the structure and physicochemical properties of plant foods significantly affect the rate and extent of nutrient digestibility [e.g., cell wall (dietary fiber) encapsulation behavior] (6, 34). For instance, to be optimally digested, lipids must be released from the cells of almond tissue and emulsified (35). In the present study, we have shown that the proportion of lipid released from the almonds following mastication is severely limited. Thus, lipid bioaccessibility values predicted by the theoretical model or determined experimentally were very low, within the range of 8–11% for almonds, with the RA form being slightly higher at the top end of this range. The greater number of small particles in the RA boluses is probably related to the reduced water content of the almond tissue, including the cell walls. Thus, because water can act as a plasticizer, the almond tissue becomes more brittle when dehydrated by roasting (36). An interesting and important observation was that the human volunteers produced a relatively high proportion of large particles after mastication, with ∼35–40% of almond particles >500 μm (some >3.35 mm; sieving data only), which explains why chewed almonds have such a low lipid bioaccessibility. This observation is consistent with the results of a digestibility study showing that the hydrolysis of lipid in almonds, albeit in 2-mm cubes rather than masticated samples, is restricted to ∼10% in the early stages of digestion (≤3 h) in the gastric and duodenal phases (7). Restricted bioaccessibility and digestion of lipid after mastication also play a crucial role in reducing postprandial lipemia (5) and may provide some explanation of why the consumption of whole almonds suppresses hunger and the desire to eat, as recently reported by the Mattes group (37). The same group had previously demonstrated the importance of chewing in relation to gut hormone signaling and the effect on satiety (9). Indeed, a bolus composed of hard, large-sized particles (>1–2 mm) delays gastric emptying because they cannot pass through the pylorus (the so-called sieving effect) (38), inducing a feeling of fullness and lower subsequent energy intake (39). However, no attempt has been made previously to characterize masticated almonds to allow quantification of lipid available for digestion, including the early stages of digestion, which is a key determinant of postprandial lipemia (5) and other metabolic responses (3).

In the present study, microstructural examination of the masticated almond tissue has shown extensive rupturing of cells at the fractured surface of the almond particles and that underneath this fractured surface are layers of intact cells retaining their intracellular contents ( ). By reducing particle size, the mastication process increases the release of nutrients from almonds, because smaller particles correspond to greater surface area to volume ratios and therefore a greater number of fractured cells. The microstructural images provide further evidence that a significant amount of almond lipid was not released by mastication, because the lipid component was still enclosed inside intact cells. This effect is strongly linked to the increase in particle size, as predicted by the theoretical model. We also found that depending on the size of the masticated particles, the degree of damage to particles varied markedly. For large particles (size >500 μm), only the cells on the fractured surface appeared to be disrupted by mastication, so that the structural integrity of cells underneath this fractured layer was much less affected. On the other hand, many smaller particles showed severe damage even in the cells located in the core of the particles.

As reported previously (19, 40), an almond bolus before swallowing consists of particles of a broad range of particle sizes, which is consistent with the multimodal PSDs obtained for both almond forms seen in the current study. One explanation for the wide size range of particles in the almond boluses has been proposed by Flynn and colleagues (41). They suggested that the mouth contains several compartments where food fracture differs. Thus, during mastication, some particles are broken into several smaller fragments, whereas others are retained in “nonmastication” compartments of the oral cavity inaccessible to the crushing or grinding action of the teeth. The adhesion of the compressed particles to the contact surfaces of the teeth while masticating probably amplifies this phenomenon and, as such, almond material adhering to teeth surfaces will be more easily fractured than freely moving particles (21). This may also explain the greater damage that occurred to the small almond particles, which has an important bearing on lipid bioaccessibility. Consequently, a greater number of fissures in the almond tissue below the fractured surface may result in an increase in the accessibility of lipid substrate to digestive fluids containing lipase and bile salts (7).

It has been suggested that the initiation of swallowing relies to some extent on a particle size threshold; however, the ready-to-swallow bolus must also be cohesive to prevent particles getting into the airways. The overall lubrication and softening of the bolus, as a result of the incorporation of saliva into the bolus, is crucial for the process of swallowing to occur (42). The sensory signals received by the mouth receptors trigger deglutition based notably on the physical properties of the bolus, such as texture, following insalivation and particle size reduction of the ingested food (42, 43). Therefore, perhaps not surprisingly and in agreement with the current data, the PSDs of almond boluses are normally similar between subjects (18, 19, 40, 42, 43), despite the fact that mastication is highly individual in terms of chewing pattern (23, 24).

As discussed, laser diffraction provided a reliable and efficient method for obtaining size information on almonds masticated by human volunteers. Compared with sieving, laser diffraction generated much more data from narrower size intervals. However, for applying size data to the theoretical model for predicting bioaccessibility, we needed additional information on the largest masticated particles (≥1.7 mm) by using the sieving method. Given the importance of mastication in influencing bioaccessibility, digestion kinetics, postprandial lipemia, and energy metabolism (5–11), we believe this novel approach of combining in vitro and in vivo methods with mathematical modeling has potential for the future. This approach could be applied, for instance, to other nutrients (e.g., starch and vitamin E) (7, 44) in plant foods, in which cell wall (fiber) rupture is the predominant mechanism of nutrient release (5–7), including nuts and seeds with similar properties to almonds.

In conclusion, we have developed a new method for determining lipid bioaccessibility of masticated almonds, showing that most lipid (∼89–92%) is retained within the tissue matrix (i.e., as intracellular lipid). An encapsulated lipid mechanism provides a plausible explanation of why almonds elicit a low postprandial lipemic response (5) and have a low metabolizable energy content despite their status as a high energy density food (8). This mechanism may also partly explain the sustained weight loss induced by an almond-enriched diet (37, 45). Almond consumption has therefore positive health implications beyond their nutritional content, including a reduction in cardiovascular disease risk factors.

## Supplementary Material

Supplemental data

## Footnotes

5 Abbreviations used: NA, natural almond; PSD, particle size distribution; RA, roasted almond.
