# Supplementary material for: Effect of mastication on lipid bioaccessibility of almonds in a randomized human study and its implications for digestion kinetics, metabolizable energy, and postprandial lipemia1
Source: Am J Clin Nutr. 2014 Nov 12;101(1):25–33. doi: 10.3945/ajcn.114.088328 (PMC4266890; doi:10.3945/ajcn.114.088328)
Supplement: Supplemental data [file supp_101_1_25__index.html]

Effect of mastication on lipid bioaccessibility of almonds in a randomized human study and its implications for digestion kinetics, metabolizable energy, and postprandial lipemia — Effect of mastication on lipid bioaccessibility of almonds in a randomized human study and its implications for digestion kinetics, metabolizable energy, and postprandial lipemia — Supplemental data 

# Effect of mastication on lipid bioaccessibility of almonds in a randomized human study and its implications for digestion kinetics, metabolizable energy, and postprandial lipemia

## Supplemental data

**Files in this Data Supplement:**

- Supplemental data - Table 1
